# Linking lipid profile alterations to antibiotic tolerance and natural product synergy in drug-resistant *Mycobacterium tuberculosis* clinical isolates

**DOI:** 10.1038/s41598-026-41967-5

**Published:** 2026-02-28

**Authors:** Anna Zabost, Rafał Sawicki, Grzegorz Jankowski, Marcin Ziomek, Wiesław Truszkiewicz, Arkadiusz Syta, Benita Hryć, Ewa Augustynowicz-Kopeć, Piotr Podlasz, Małgorzata Chmielewska-Krzesińska, Elwira Sieniawska

**Affiliations:** 1 Department of Microbiology, National Tuberculosis and Lung Diseases Research Institute, Warsaw, Poland; 2 Chair and Department of Biochemistry and Biotechnology, Medical University of Lublin, Lublin, Poland; 3 Department of Natural Products Chemistry, Medical University of Lublin, Chodźki 1, Lublin, 20-093 Poland; 4 Doctoral School of the Medical University of Lublin, Chodźki St. 7, Lublin, 20-093 Poland; 5 Department of Technical Computer Science, Lublin University of Technology, Lublin, Poland; 6 Department of Pathophysiology, Forensic Veterinary Medicine and Administration, Faculty of Veterinary Medicine, University of Warmia and Mazury in Olsztyn, Olsztyn, Poland

**Keywords:** Lipidomics, LC-MS, Natural products, Piperine, Thymoquinone, Drug discovery, Microbiology

## Abstract

Despite global control efforts, tuberculosis remains the leading infectious cause of death, with rising incidence, pediatric cases, and drug-resistant strains posing major public health challenges. Mycobacteria, including *Mycobacterium tuberculosis*, possess a lipid-rich, dual-membrane cell envelope that contributes to their impermeability, drug resistance, and unique pathogenic mechanisms. Some lipids play key roles in modulating host immune responses, enabling survival within macrophages, and promoting granuloma formation. Since it is known that lipid remodeling of the cell envelope is correlated with the antibiotics tolerance in mycobacteria we used liquid chromatography coupled to mass spectrometry to analyze the lipid profiles of *M. tuberculosis* clinical isolates with diverse drug-resistance characteristics in order to investigate if there is any link between Mtb lipids composition, its drugs susceptibility and the antimycobacterial activity of natural small molecules used in combination with first line antibiotics. The results showed that among cross combinations of antibiotics and natural products (piperine and thymoquinone) the potentiation of antimycobacterial activity was obtained in all strains only for rifampicin. Drug-resistant isolates presented the shift in glycerophospholipids building the inner membrane towards molecules with shorter acyl chains, but the decreased membrane hydrophobic thickness was compensated in some strains by increased membrane rigidity. The pXDR/XDR isolates accumulated mycobactins loaded iron and showed dysregulation in the production of phthiocerol/phthiodiolone dimycocerosates and triacylglycerols.

**Supplementary Information:**

The online version contains supplementary material available at 10.1038/s41598-026-41967-5.

## Introduction

Mycobacteria (including *Mycobacterium tuberculosis*) are characterized by a unique lipid profile, which affects their physiology, pathogenicity and resistance. Lipids account for up to 60% of the mycobacteria dry cellular weight, and up to 40% of the cell envelope, therefore although belonging to Gram-positive bacteria they are faintly stained^1,2^. Hydrophobicity, hence impermeability of the mycobacterial cell envelope for dye and antibiotics, is attributed to its binary membrane structure: outer and inner membrane. The outer membrane (OM) is composed of mycolic acids (so-called mycomembrane) covalently linked with arabinogalactan, and external non-covalently attached lipids. Inner membrane (IM) is formed from glycerophospholipids: diacylglycerophosphoinositols (PI), diacylglycerolphosphoethanolamines (PE), cardiolipins (CL), and a large amount of acylated diacylglycerophosphoinositols mannosides (AcPIMs). Between both membranes, the arabinogalactan–peptidoglycan complex (attached to mycolic acids), and the periplasmatic space filled with lipoglycans: lipomannan (LM) and lipoarabinomannan (LAM), anchored in IM are found^1,3,4^. Some lipids, like diacylglycerophosphoinositols manosides (PIMs), AcPIMs, LM, LAM, mycolic acids, or trehalose-containing glycolipids are present only within members of the genus Mycobacteriaceae and related bacteria in the Actinobacteria phylum^5–7^.

Besides forming a physical barrier, cell envelope lipids play a crucial role in *M. tuberculosis* (Mtb) pathogenicity in tuberculosis (TB). Phenolic glycolipids (PGLs), trehalose dimycolates (TDM), saccharolipids, phthiocerol dimycocerosates (PDIM), mycolic acids, and lipoglycans can either trigger or suppress immune responses, depending on the phase of infection^8^. LAM interferes with macrophage and dendritic cells function by inactivating tyrosine kinases, blocking calcium signaling, and impairing phagosome maturation. These lead to avoidance of lysis and antigen presentation. LM modulates cytokine production (e.g. inducing IL-12)^9^. TDM interacts with host cell receptors (C-type lectin receptors Mincle) to promote the bacteria’s entry into host cells and modulates the immune response, allowing for bacterial replication^10,11^. Moreover, TDM stronger than LAM and PIMs induces pulmonary granuloma formation through an IFN-γ-independent and TNF-α-dependent pathway^12^. PGLs suppress the release of cytokines involved in the inflammatory response^13^, whereas saccharolipids block phagosome maturation, inhibit T cell proliferation and down-regulate cytokine secretion in activated monocytes^14–16^. Also, PDIM contribute to the ability of mycobacteria to escape the phagosome through membrane disrupting activity and induction of necrosis mediated by the increased reactive species production^17,18^. Finally, mycolic acids possess varying capabilities to induce foamy macrophages and trigger immune responses^19^. Such an arsenal of active lipid molecules makes *M. tuberculosis* difficult to eradicate and tuberculosis challenging to eliminate.

Lipid remodeling of the mycobacterial cell envelope is increasingly recognized as an important contributor to antibiotic tolerance, as it influences membrane permeability, drug transport, and the activity of resistance-associated systems. Recent studies have shown that adaptive changes in lipid composition and membrane dynamics promote phenotypic tolerance in *Mycobacterium tuberculosis* independently of classical resistance mutations^20^,^21^. Outer envelope lipids such as phthiocerol dimycocerosates (PDIM) have been specifically implicated in stress- and starvation-induced antibiotic tolerance, highlighting the functional importance of lipid homeostasis under adverse conditions^22^. Moreover, membrane lipid composition can modulate the activity of multidrug efflux transporters^23^, which is particularly relevant for *M. tuberculosis*, where the Rv1258c (Tap) efflux pump contributes to resistance to multiple first-line drugs^24,25^. These findings establish lipid remodeling as a key adaptive strategy shaping antibiotic tolerance and resistance in mycobacteria. We therefore examined lipidomic profiles of drug-susceptible and drug-resistant *M. tuberculosis* clinical isolates to assess their association with antibiotic susceptibility and the activity of natural compounds used as adjuvants to first-line drugs.

## Results

### Susceptibility profile of panel strains

The experimental panel was composed to represent strains with different drug susceptibility. The characterization of investigated strains is presented in Table 1.

**Table 1:** Characterization of investigated strains.

Strain	Resistance pattern	Spoligotype family
H37Rv	Susceptible	H_37_Rv
1890	Susceptible	T1-RUS2 1173
1461	Susceptible	T1 53
262/19	RMP	770000403740371
235/18	RMP	T1 65
33/19	INH	LAM9 42
1183	INH	UNK SIT237
221/21	MDR	Beijing1
158/20	MDR	T1 1558
5880	pXDR	Beijing 265
2373	pXDR	Beijing 265
5228	XDR	Beijing 265
2240/21	XDR	Beijing 265

RMP, resistant to rifampicin; INH, resistant to isoniazid; MDR, multidrug resistant; pXDR, pre-extensively drug-resistant; XDR, extensively drug-resistant.

**Fig. 1:**
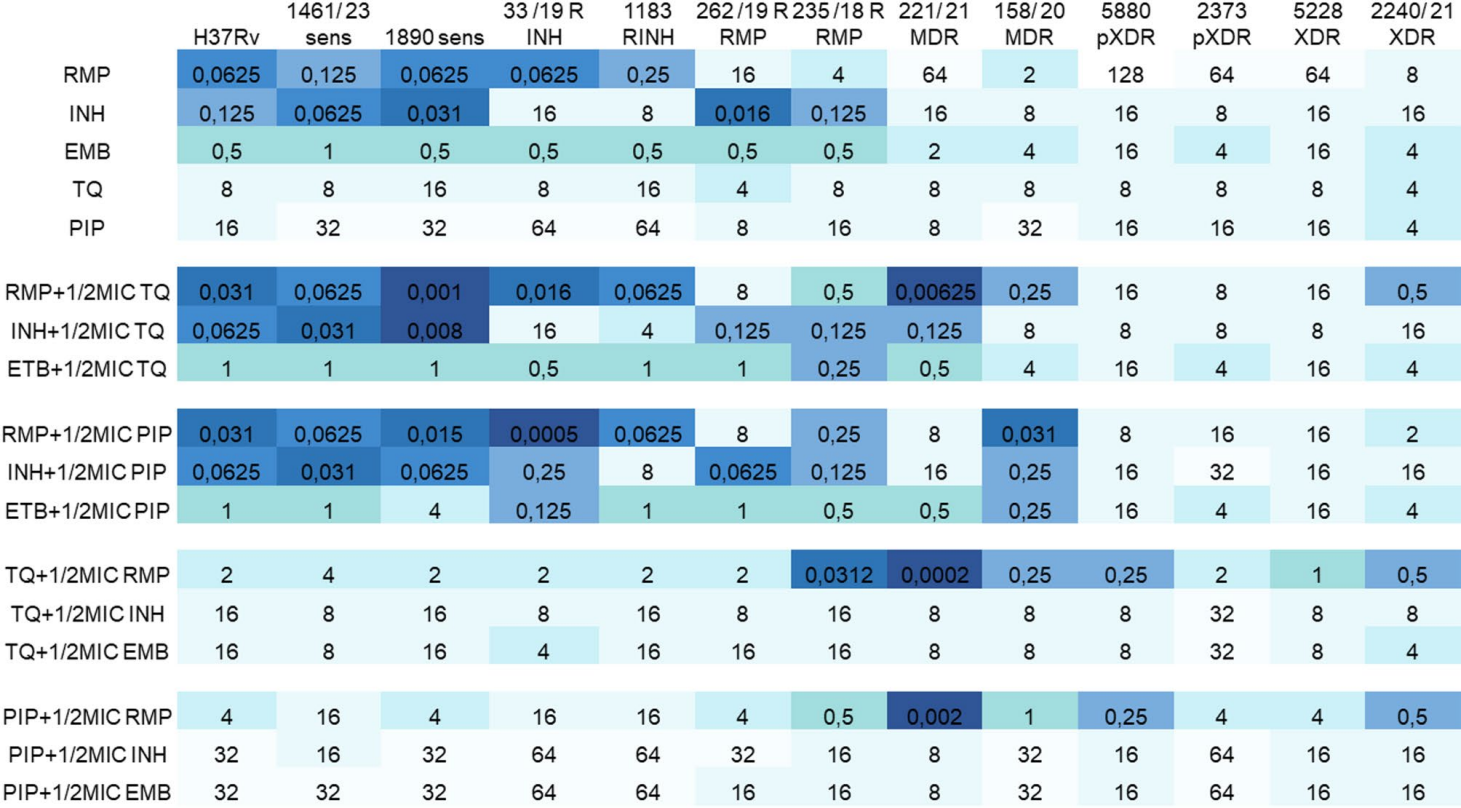
The susceptibility profile of panel strains expressed as MIC values (µg/ml) for individual substances and their combinations. RMP, rifampicin; INH, isoniazid; EMB, ethambutol; TQ, thymoquinone; PIP, piperine.

Reference H37Rv, and 1461, 1890 isolates were drug-sensitive. Minimal inhibitory concentration (MIC) values observed for the first line antimycobacterial antibiotics were the lowest for these strains (Fig. 1). MIC values of INH against isolates 33/19 and 1183, as well as MIC values of RMP against isolates 262/19 and 235/18 correlated with their resistance profiles. Accordingly, MDR, pXDR and XDR strains showed resistance to antibiotics expressed in a wide range of MIC values (2–128 µg/ml). The susceptibility of panel strains to thymoquinone (TQ) and piperine (PIP) was less diverse. MIC values were covered by three two-fold serial dilution steps (4–16 µg/ml) for TQ, and five two-fold dilution levels (4–64 µg/ml) for PIP, with lower MIC values observed against resistant strains in the case of PIP. Activity of TQ was similar to the activity of isoniazid (INH) against resistant isolates (MIC between 8 and 16 µg/ml). The combination of antibiotics and natural products (added in the concentration equal to their ½ MIC) resulted in lowering the MIC of rifampicin (RMP) and INH. The increased activity of RMP was observed against all strains in the presence of TQ and PIP. Also, the activity of INH was higher in combination with TQ and PIP, however, not against all isolates. Natural products did not affect activity of ethambutol (EMB). When natural products were supplemented with antibiotics (added in the concentration equal to their ½ MIC), their activity was improved only in the presence of RMP. Addition of INH or EMB did not have a positive effect (Fig. 1).

The highest increase in activity of RMP in combinations with TQ was observed for 221/21 MDR isolate (10240-fold), followed by 1461 sensitive strain (62-fold). The activity of RMP in combination with TQ against other strains was potentiated in the range: 2–16 times. Also the activity of INH in the presence of TQ was significantly increased against 221/21 MDR strain (125-fold). The addition of PIP to RMP resulted in a significant increase in activity against 33/19 R INH strain (125-fold) and 158/20 MDR isolate (64-fold). Moreover, PIP restored the sensitivity of 33/19 R INH strain to INH (64-fold increase in activity). When TQ and PIP were supplemented with RMP, their activity was significantly potentiated against 235/18 RMP, 221/21 MDR, 158/20 MDR, 5580 pXDR (fold change from 32 to 4000) and to a lesser extent against all other strains (2–8 times).

Because the antimycobacterial activity of the tested compounds was in a wide range of concentrations, and the significant increase in activity of compound combinations was limited to some isolates, in the next step, we analyzed the lipid profile of panel strains.

### Lipid profile of panel strains

**Fig. 2:**
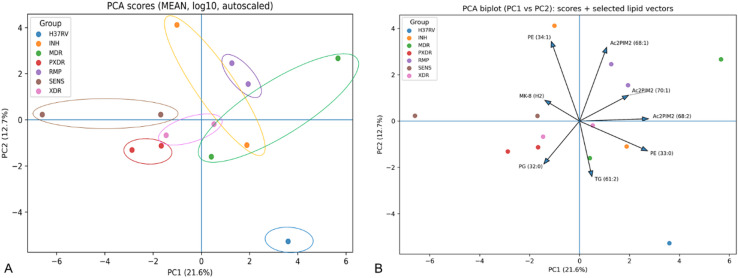
Principal component analysis (PCA) of lipidomic profiles of *M. tuberculosis* strains with different drug-resistance phenotypes. (**A**) PCA score plot based on mean-centered, log_10_-transformed and autoscaled lipidomic data (z-score standardization; mean-centered and divided by the standard deviation of each variable), performed on a feature matrix comprising mean signal intensities for 41 lipid features measured across 13 samples, showing the distribution of samples along PC1 (21.6% of explained variance) and PC2 (12.7%). Each point represents an mean from biological replicates, colors indicate resistance groups. The reference strain H37Rv is shown separately and does not cluster with any resistance group. (**B**) PCA biplot (PC1 vs. PC2) displaying sample scores together with selected lipid loading vectors. Arrows indicate lipid species contributing most strongly to sample separation, with vector direction and length reflecting the correlation and magnitude of contribution to the principal components.

To evaluate natural relationships among samples based on lipidomics profiles, we performed principal component analysis (PCA). The first three principal components explained 46.8% of the total variance (PC1 = 21.6%, PC2 = 12.7%, PC3 = 12.5%). As shown in Fig. 2, the PCA score plot revealed a partial but biologically meaningful separation of strains according to drug-resistance status. Clinical isolates exhibited distinct clustering patterns, whereas the reference strain H37Rv did not co-cluster with any resistance group and was positioned separately in the PCA space (Fig. 2A). PC1 primarily discriminated strains based on lipid species associated with cell envelope composition. Multidrug-resistant (MDR) isolates were shifted towards positive PC1 values and were strongly associated with acylated phosphatidylinositol mannosides Ac2PIM2 (68:2) and Ac2PIM2 (70:1), as indicated by the direction and magnitude of the corresponding loading vectors (Fig. 2B). In contrast, drug-sensitive (SENS) strains were located at negative PC1 values and correlated with menaquinone MK-8 (H2), suggesting preservation of a lipid profile characteristic of energetically active, non-adapted cells. The reference strain H37Rv occupied an intermediate position along PC1 but did not align strongly with any lipid vector, indicating the absence of dominant lipidomic features associated with either resistance or clinical sensitivity phenotypes. PC2 reflected differences related to metabolic adaptation and lipid storage. Isoniazid-resistant (INH) and rifampicin-resistant (RMP) strains clustered at positive PC2 values and were associated with phosphatidylethanolamine PE (34:1) and Ac2PIM2 (68:1), consistent with an increased contribution of more unsaturated membrane lipids. In contrast, XDR, pXDR, and partially MDR strains were shifted towards negative PC2 values and correlated with triacylglycerol TG (61:2) and phosphatidylglycerol PG (32:0), indicating enhanced accumulation of storage lipids. H37Rv displayed strongly negative PC2 values but remained spatially separated from XDR and pXDR clusters (Fig. 2), suggesting that although the reference strain shares low PC2 scores, it does not exhibit the same lipid remodeling patterns characteristic of highly drug-resistant clinical isolates. The PCA analysis demonstrates that drug-resistant clinical isolates undergo distinct lipidomic remodeling involving both cell envelope-associated glycerophospholipids and lipid storage pathways, whereas the laboratory reference strain H37Rv maintains a distinct lipidomic profile that does not reflect clinical resistance-associated adaptations. The lack of co-clustering of H37Rv with any resistance group highlights fundamental differences between laboratory-adapted and clinically evolved *M. tuberculosis* strains (Fig. 2).

### Global distribution of major glycerophospholipid classes

**Fig. 3:**
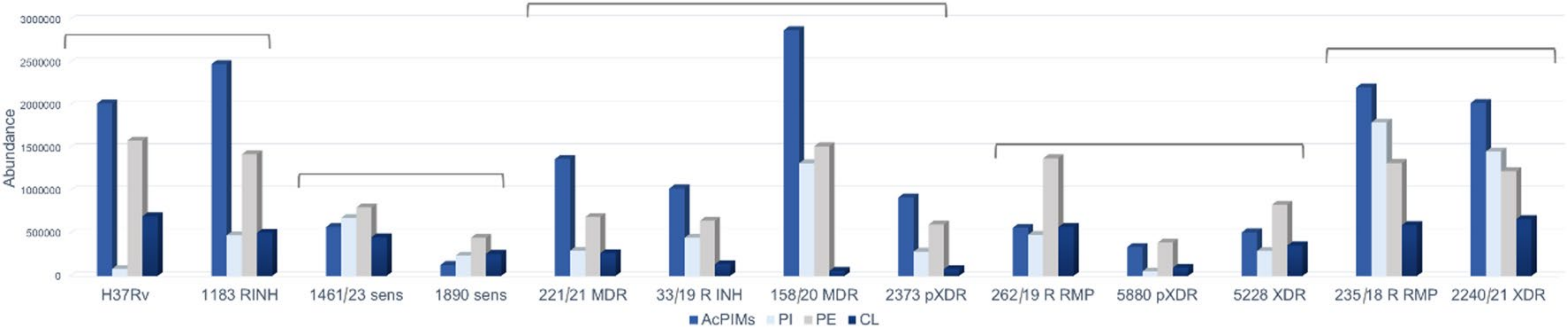
Cumulative abundance of four glycerophospholipid classes contributing to *Mycobacterium tuberculosis* inner membrane composition (AcPIMs, PI, PE, CL) across the analyzed isolates (H37Rv and clinical isolates with different drug-resistance phenotypes). Values represent the mean of three measurements per isolate. To evaluate differences across phenotypic resistance groups (SENS, INH, RMP, MDR, pXDR, XDR), we applied the Kruskal–Wallis test on log10-transformed values with Benjamini–Hochberg FDR correction. No significant differences were detected for any lipid class: AcPIMs *p* = 0.4158 (q = 0.4158), PI *p* = 0.3128 (q = 0.4158), PE *p* = 0.2349 (q = 0.4158), CL *p* = 0.1645 (q = 0.4158) (all q > 0.05); AcPIMs, acylated phosphatidylinositol mannosides; PI, phosphatidylinositols; PE, phosphatidylethanolamines; Cl, cardiolipins; Brackets indicate groups of strains exhibiting similar trends in the relative proportions of these lipid classes.

The overall abundance of the major glycerophospholipid classes: acylated phosphatidylinositol mannosides (AcPIMs), phosphatidylethanolamines (PE), phosphatidylinositols (PI), and cardiolipins (CL), varied substantially across strains (Fig. 3). The relative proportions of these lipid classes only partially reflected drug-resistance status, but clear grouping patterns emerged based on the dominance of AcPIMs or PE. Strains characterized by AcPIMs dominance generally exhibited a higher overall glycerophospholipid abundance compared with those in which PE predominated. In sensitive clinical isolates, PI tended to be more abundant than AcPIMs, whereas in MDR/XDR strains AcPIMs dominated over PI, consistent with their positive shift along PC1.

The detailed molecular composition of phosphatidylethanolamines, cardiolipins and phosphatidylinositols revealed pronounced strain-specific differences. Across all strains, the major phosphatidylethanolamine species were PE (32:0), PE (34:0), PE (35:0), and PE (34:1). Saturated PE species predominated, followed by species containing one double bond, with only a minor contribution from more unsaturated forms. The relative contribution of monounsaturated PE species was higher in all clinical isolates compared to H37Rv, with selected strains showing enrichment of PE (34:1). These patterns are in agreement with the association of PE-related features with PC2. Detailed distributions of PE carbon number and unsaturation are shown in Supplementary Fig. S1A, S2A,B. Cardiolipins displayed the highest molecular diversity among glycerophospholipids. CL(68:2) was detected in all sensitive and single drug-resistant strains, whereas shorter-chain CL species (60–66 carbons) were enriched in MDR/XDR isolates. Differences were also observed in the degree of unsaturation, with MDR/XDR strains showing increased proportions of CL species containing either zero or three double bonds. Detailed CL profiles are presented in Supplementary Fig. S1B, S2C,D. Phosphatidylinositol composition further distinguished the reference strain from clinical isolates. In H37Rv, PI species with 35–37 carbons predominated, whereas clinical isolates showed a more heterogeneous distribution, including increased abundance of shorter PI species (32–33 carbons) in resistant strains. Highly unsaturated PI species were largely absent from resistant isolates, with only sporadic exceptions. Detailed PI distributions are shown in Supplementary Figure S1C, S2E,F.

### Acylated phosphatidylinositol mannosides as key discriminators

**Fig. 4:**
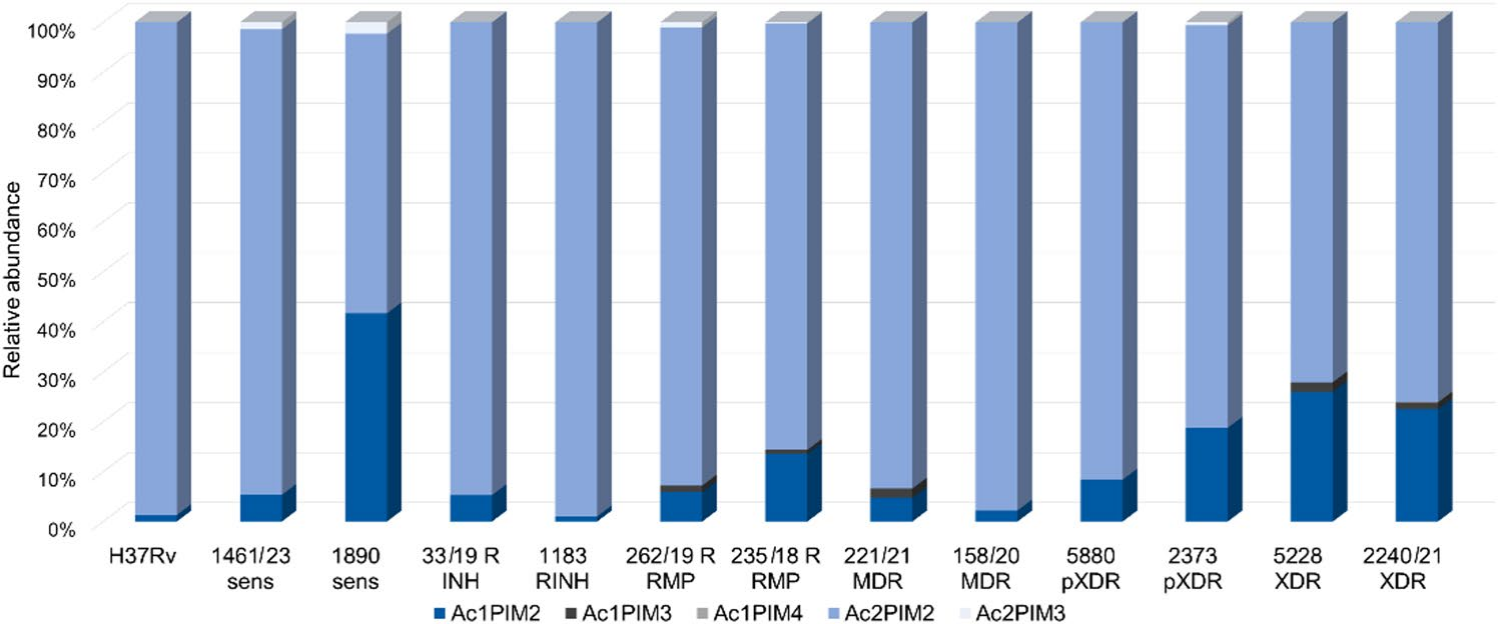
Acylated diacylglycerophosphoinositol mannosides (AcPIMs) composition in the panel strains. Relative abundance represents the contribution of individual lipids to the whole subclass. A—AcPIMs by number of acyl chains and mannose units; Relative abundance was calculated as the proportion of each individual lipid relative to the total abundance of all lipids within the same subclass, such that the cumulative abundance of the subclass equals 100%.

Acylated phosphatidylinositol mannosides constituted a central discriminatory lipid class across all strains. Doubly acylated dimannosides (Ac2PIM2) predominated universally, with Ac2PIM2 (71:1) and (71:2) as the most abundant species (Fig. 4). However, while H37Rv consisted almost exclusively of Ac2PIM2, clinical isolates exhibited greater diversity, including the presence of Ac1PIM2 and Ac1PIM3 species, particularly in RMP-resistant, MDR, pXDR, and XDR strains. Resistant isolates were characterized by shorter and more saturated AcPIM acyl chains, whereas sensitive strains generally showed enrichment of longer and more unsaturated species, in agreement with their separation along PC1. Detailed distributions of AcPIM chain length, unsaturation and the ranked abundance of AcPIM molecular species is shown in Supplementary Fig. S3.

### Neutral lipids and mycobactins

Neutral lipid classes, including mono-, di- and triacylglycerols (MG, DG and TG), were detected in all strains. DGs predominated in most isolates, whereas TG accumulation was enhanced in selected pXDR and XDR strains. Detailed quantitative distributions, including fatty acid parity and strain-specific variability, are provided in Supplementary Fig. S4.

Mycobactins lacking iron were detected in all analyzed strains, whereas iron-loaded mycobactins were observed only in selected pXDR and XDR isolates. Quantitative profiles and representative mass spectra are shown in Supplementary Fig. S5.

In addition to glycerophospholipid remodeling, variability was also observed in mycolic acid subclasses and PDIM-related molecules across strains; however, these differences were heterogeneous and largely qualitative in nature (Supplementary Fig. S6).

In the profile of mycolic acids, the lack of molecules with the longest chains (87–90 carbons) was observed in clinical strains (Supplementary Fig. S6). An even more pronounced alteration was detected for phthiocerol dimycocerosates (DIMA) and phthiodiolone dimycocerosates (DIMB) in drug-resistant isolates. RINH strains, the 5228 XDR isolate, and both pXDR strains showed an almost complete absence of DIMB. Similarly, 5228 XDR and 2373 pXDR lacked all detectable DIMA, whereas DIMA species with very long chains was also absent in 221/21 MDR, 2240/21 XDR, and 5880 pXDR isolates. Interestingly, the lack of long chains DIMA (97–100 carbons) was also observed for the 1980 sensitive isolate (Supplementary Fig. S6). These observations indicate heterogeneous remodeling of outer-envelope lipids that accompanies, but does not directly drive, the multivariate lipidomic patterns identified by PCA.

## Discussion

The present study demonstrates that the natural compounds piperine (PIP) and thymoquinone (TQ) selectively potentiate the activity of rifampicin (RMP) and isoniazid (INH), but not ethambutol (EMB), against *Mycobacterium tuberculosis* clinical isolates. RMP inhibits RNA polymerase, INH inhibits mycolic acid synthesis (cell wall), whereas EMB targets arabinosyl transferases, involved in arabinogalactan biosynthesis^26^. Although both INH and EMB interfere with cell wall assembly, the fatty acid synthase II (FAS-II) system targeted by INH is cytoplasmic and membrane-associated^27^, whereas arabinosyltransferases inhibited by EMB are membrane-embedded enzymes located in the periplasmic space^28^. The lack of EMB potentiation therefore suggests that PIP and TQ primarily affect cytoplasmic resistance mechanisms, potentially through modulation of drug efflux or intracellular metabolic processes. PIP restored susceptibility to INH in one INH-resistant strain and enhanced RMP activity across all tested isolates. Previous studies demonstrated that PIP inhibits the Rv1258c efflux pump, which is frequently overexpressed in RMP-resistant Mtb, leading to increased intracellular antibiotic accumulation^25^. This mechanism likely explains the improved activity of RMP observed in all strains and the strain-dependent improvement in INH susceptibility, as the extent of efflux inhibition depends on the baseline efflux activity^24^. In addition, computational docking studies suggested that PIP may interact with essential domains of RNA polymerase^29^ potentially augmenting the inhibitory effect of RMP on transcription, targeting β-subunit of bacterial DNA-dependent RNA polymerase^30^. The combined inhibition of efflux and transcription may therefore underlie the pronounced efficacy of the PIP–RMP combination, particularly in resistant isolates. In contrast, TQ exhibited comparable antimycobacterial activity across all strains, regardless of resistance status. Previously, we demonstrated that TQ induces depletion of intracellular ATP and NAD pools in *M. tuberculosis*, leading to metabolic collapse and impaired lipid synthesis^31^. The enhanced activity of RMP observed upon TQ co-treatment may therefore arise from additive metabolic stress, whereby ATP and NAD depletion compromises the ability of bacteria to sustain transcriptional responses and repair mechanisms following RMP-mediated inhibition of RNA polymerase, which affects respiration/metabolism in ways that interact with energy state^32^. Additionally, TQ-induced disruption of lipid synthesis and energy metabolism may indirectly reduce the activity of energy-dependent efflux systems, further increasing intracellular antibiotic exposure.

Lipidomic profiling revealed substantial remodeling of membrane-associated lipids among clinical isolates, partially reflecting their drug susceptibility profiles. All strains were cultivated under optimal growth conditions and harvested during the late logarithmic phase, to minimize variability related to growth stage–dependent lipid remodeling. Despite this, clinical isolates displayed marked differences in the relative abundance of glycerophospholipid classes compared with the laboratory reference strain H37Rv, indicating that strains circulating in patients undergo distinct metabolic adaptations. In several isolates, phosphatidylethanolamine (PE) dominated over acylated phosphatidylinositol mannosides (AcPIMs), whereas in others, including H37Rv, AcPIMs remained the most abundant class, highlighting heterogeneity in membrane organization. Ac2PIM2 species constituted the majority of AcPIMs in all isolates, and nearly the entire AcPIM pool in H37Rv and one INH-resistant strain. In contrast, Ac1PIM2 species, which contain fewer acyl chains, were more prevalent in multiple resistant isolates as well as in one clinically sensitive strain, underscoring differences between laboratory-adapted and patient-derived bacteria.

Strains in which AcPIMs were not prevalent overproduced PE. Previous studies in *Mycobacterium smegmatis* reported that cardiolipin (CL), PE, and PI/PIMs constitute approximately 37%, 32%, and 28% of the total phospholipids in the plasma membrane, respectively^33^. However, PE predominance has not been described previously. Strains characterized by reduced AcPIM abundance frequently overproduced PE, although the functional basis for prioritization of PE synthesis over AcPIMs and CL remains unclear. Nevertheless, these shifts suggest coordinated regulation among major membrane lipid classes rather than random variation.

A prominent lipidomic feature associated with MDR and XDR strains was the shortening of acyl chains in CL, PI, and AcPIMs. In contrast, PE profiles remained relatively conserved across all strains, indicating that PE may play a limited role in adaptive membrane remodeling. Alterations in acyl chain saturation were also observed, although these changes were strain-specific rather than uniform. Increased saturation of PI and AcPIMs was particularly evident in isolates in which PE dominated the membrane lipid composition, suggesting compensatory adjustments in membrane rigidity when PE abundance increases. This suggests that since PE did not impact the IM stability maintenance, the compensatory modification (increased saturation) was made in lipids with an inositol head group. Although shorter acyl chains usually mean a more fluid, leakier membrane, making bacteria more sensitive, more saturated fatty acids contribute to straighter chains packed more tightly in the membrane, increasing rigidity and hydrophobicity^34^. These findings indicate that resistant isolates adapt their membrane properties through coordinated modulation of lipid chain length and saturation rather than changes in a single lipid class. Such remodeling may reflect compensatory responses to antibiotic pressure. Mutations in β-ketoacyl synthase I (KasA), a key enzyme in mycolic acid synthesis, have been described in INH-resistant *M. tuberculosis*. Although kasA overexpression has been reported, mutant KasA proteins produce mycolic acid precursors less efficiently than the wild-type enzyme^35^. Consistent with these observations, resistant strains in the present study lacked mycolic acids with the longest carbon chains (87–90 carbons), supporting the notion of compensatory remodeling of lipid biosynthesis pathways. Shortening of acyl chains may reduce metabolic costs or reliance on inhibited pathways, while increased saturation may counterbalance the resulting increase in membrane fluidity, thereby limiting antibiotic penetration.

Phthiocerol dimycocerosates (DIM A and DIM B) are mycolic acid–derived lipids characteristic of the outer cell envelope of *M. tuberculosis*^36^. The complete lack of DIM A and DIM B in two pXDR/XDR strains, and their marked reduction in one pXDR isolate suggests an additional adaptive response that may influence bacterial physiology. Although loss of DIM has been associated with increased cell envelope permeability and enhanced drug susceptibility^37^ this phenotype was not accompanied by increased in vitro sensitivity to first-line drugs in the present study, indicating that DIM depletion does not necessarily confer drug sensitivity. Loss of DIM production has been linked to disruptions in PDIM biosynthesis^38^ or to metabolic constraints affecting methylmalonyl-CoA availability, a key precursor for DIM synthesis^39^. Especially, isolates lacking DIM A/B exhibited pronounced accumulation of triacylglycerols, often enriched in odd-chain fatty acids, suggesting rerouting of excess propionyl-CoA into neutral lipid storage. This metabolic shift is consistent with the PCA-driven separation of highly resistant strains along PC2, which was strongly associated with storage lipid accumulation, and highlights an alternative adaptive strategy in strains with compromised PDIM biosynthesis.

Changes in mycobactin abundance were observed in selected pXDR/XDR isolates; however, these metabolites did not contribute significantly to PCA separation. This indicates that iron-scavenging adaptations, while potentially relevant for oxidative stress tolerance, represent secondary, strain-specific responses rather than dominant determinants of lipidomic variance.

In summary, our results demonstrate that PIP and TQ enhance the activity of rifampicin and isoniazid through mechanisms that likely involve inhibition of efflux, disruption of transcription, and metabolic destabilization. Drug-resistant *M. tuberculosis* isolates exhibit extensive lipidomic remodeling, characterized by altered lipid class ratios, shortened acyl chains, and changes in saturation, which together may modulate membrane properties and drug transport. The loss of DIM A/B and accumulation of TG further indicate profound metabolic reprogramming in highly resistant strains. These findings highlight lipid metabolism and membrane adaptation as integral components of antibiotic resistance and support the potential of natural compounds such as PIP and TQ as adjuvants targeting these processes.

## Materials and methods

### Bacterial strains used in this study

The tuberculostatic activity of natural products was tested in the Department of Microbiology, National Tuberculosis and Lung Diseases Research Institute, Warsaw, Poland. The compounds were examined in vitro for their tuberculostatic activity against the *M. tuberculosis* H37Rv strain (ATCC 25618), twelve “wild” strains isolated from TB patients: two (1890, 1461) fully sensitive to the administrated tuberculostatics, two (262/19, 235/18) resistant to rifampicin (RMP), two (33/19, 1183) resistant to isoniazid (INH), two (221/21, 158/20) resistant to RMP, INH (MDR), two (5880, 2373) resistant to RMP, INH and fluoroquinolones (FLQ), two (5228, 2240/21) resistant to RMP, INH, FLQ and linezolid. The strains were isolated according to routine procedures from patients suspected of TB and were subjected to typical TB diagnostics (identification and drug resistance). The strains for testing were randomly selected from the TB mycobacterium strain bank maintained by the National Reference Laboratory of Mycobacteria. The criterion for selecting strains was the drug resistance profile. The spoligotyping was performed following the manufacturer’s instructions (Mapmygenome Diagnostics, Hyderabad, Telangana, India).

### *In vitro* antitubercular activity

The experiments were carried out in sterile 96-well microtiter plates using a twofold serial dilution method in Middlebrook 7H9 broth (Becton, Dickinson and Company, Franklin Lakes, NJ, USA) supplemented with 10% Middlebrook oleic albumin dextrose catalase (OADC; Becton, Dickinson and Company, Franklin Lakes, NJ, USA). For inoculum preparation, the bacterial culture was suspended in 5 mL of 7H9 medium and vortexed with glass beads for 3 min. The suspension was allowed to settle for 30 min at room temperature, after which the supernatant was transferred to a sterile tube and left undisturbed for an additional 15 min. One milliliter of the clarified supernatant was adjusted to a 0.5 McFarland turbidity standard using OADC-enriched 7H9 broth, then diluted 1:100 to yield a final bacterial concentration of 1.5 × 10^7^. The same inoculum preparation protocol, incubation conditions, and MIC readout criteria were applied uniformly across all strains and experiments. Stock solutions of the tested natural compounds — thymoquinone (TQ; Cayman Chemicals, Ann Arbor, MI, USA) and piperine (PIP; Merck KGaA, Darmstadt, Germany) —were prepared in dimethyl sulfoxide (DMSO, Merck KGaA, Darmstadt, Germany) at 1024 µg/mL. These were further serially diluted in the 96-well plates with 100 µL of OADC-supplemented 7H9 medium. Subsequently, 100 µL of the bacterial suspension was added to each well, resulting in a final inoculum of approximately 5 × 10^5^ CFU/mL. The final test concentrations of the compounds ranged between 0.25 and 512 µg/mL, while the DMSO content never exceeded 2% (v/v). Each plate included a growth control (no drug) and a sterility control (no inoculum). Plates were incubated at 37 °C for 3 weeks. Following incubation, 30 µL of Alamar Blue (Merck KGaA, Darmstadt, Germany) solution was added to each well, and plates were incubated for another 24 h. A color shift from blue to pink indicated bacterial growth. The minimum inhibitory concentration (MIC) was defined as the lowest drug concentration that prevented the color change. For comparison, standard anti-tuberculosis drugs: isoniazid (INH), rifampicin (RMP), and ethambutol (EMB) (Merck KGaA, Darmstadt, Germany) were included as reference controls. Combinations of the tested natural products and the reference drug were prepared in Middlebrook 7H9 medium (Becton-Dickinson, USA) supplemented with 10% OADC (Becton-Dickinson, USA) in 96-well microtiter plates, following the same procedure as for MIC determination. Each compound in concentrations ranging from 1 to $\:1/32$ MIC was mixed with the reference drug in a concentration equal to 1/2 MIC. The use of 1/2 MIC concentrations was selected to enable detection of potentiation effects without complete growth inhibition by either compound alone, allowing comparative assessment of adjuvant activity across strains. Simultaneously, each of the reference drugs (isoniazid, rifampicin and ethambutol) in concentrations ranging from 1 to 1/32 MIC was mixed with the tested derivative in concentration equal to 1/2 MIC (MIC values of tested derivatives and reference drugs alone were assigned as described above). All MIC determinations and combination assays were performed using independent biological cultures prepared on separate days, with each condition tested in at least three biological replicates. Growth, sterility, and solvent controls were included in each experiment.

### Lipids analysis

Total lipids were extracted from 50 mg samples of lyophilized bacteria with a mixture of chloroform: methanol (2:1v/v; 1.5 mL) (Folch method). Samples were sonicated for 20 min (without heating) and centrifuged (15 min at 12,700 rpm at 4 °C). Then, the supernatant was collected and the extraction procedure was repeated. Combined supernatants were evaporated under reduced pressure at 30 °C. Extracts were dissolved in 1 ml of the mixture of hexane-isopropanol (70:30 v/v), filtered through the PTFE syringe filters (0.22 μm), and 10 µL was injected into the Reprospher 100 Diol chromatographic column (3 μm, 150 × 2 mm; Dr Maich GmbH, Ammerbuch, Germany) set at 20 °C. Separation was performed on Agilent 1200 Infinity HPLC (Agilent Technologies, Santa Clara, CA, USA) under the following elution gradient of solvent A (hexane/isopropanol, 70:30, v/v) and solvent B (isopropanol/methanol, 30:70, v/v): 0% B for 10 min, 0–50% B for 7 min, 50% B for 5 min, 50–100% B for 8 min and 100% B for 10 min with 10 min of post-run time at initial conditions. The mobile phase flow rate was 0.15 mL/min. The mass spectrometry (MS) data acquisition was performed on Agilent 6530B QTOF Accurate-Mass QTOF spectrometer working with the Dual Agilent Jet Stream Electrospray ionization source (Dual AJS ESI) (Agilent Technologies, Santa Clara, CA, USA) operating in positive and negative ionization mode. The MS acquisition parameters were as follows: drying gas temperature: 325 °C; drying gas flow: 8 L/min; nebulizer pressure: 35 psig; sheath gas temperature: 350 °C; sheath gas flow: 10 L/min; capillary voltage: 5500 V; Fragmentor: 50 V; skimmer: 65 V. The MS and MS/MS acquisition m/z ranges were 100–3200 and 50–3200, respectively. Collision energies were set at 30 and 60 eV. The analysis was performed in three replicates.

Mycolic acids and DIMA/B methyl esters were analyzed after hydrolysis and methylation procedure adopted from Sambandan et al.^40^. Lyophilized bacteria (samples of 50 mg) were placed in glass tubes and mixed with 2 mL of water. Then, 2 mL of 40% tetrabutylammonium hydroxide was added and the suspension was heated at 100 °C for 20 h. When samples were cooled down, 200 µL of iodomethane and 4 mL of dichloromethane were added, followed by shaking at room temperature for 1 h. The collected organic phase was washed with 2 mL of 1 M HCL and 2 mL of water, then evaporated. 400 µL of dichloromethane was used to dissolve the residue. 10 µL of the sample was injected into Reprospher 100 Diol chromatographic column (3 μm, 150 × 2 mm; Dr Maich GmbH, Ammerbuch, Germany) (20 °C) under the following elution program: gradient of solvent A (hexane/isopropanol, 70:30, v/v) and solvent B (isopropanol/methanol, 30:70, v/v) as follows: 0% B for 20 min, 0–100% B for 5 min, 100% B for 5 min with 15 min of posttime; flow rate was 0.15 mL/min. Metabolites were ionized in the positive ion mode with the same MS detection conditions as described for other lipids. Analysis was performed on Agilent 1200 Infinity HPLC coupled to Agilent 6530B QTOF working with Dual Agilent Jet Stream ESI (Agilent Technologies, Santa Clara, CA, USA). The analysis was performed in three replicates.

Quality control for LC-QTOF-MS analyses included continuous monitoring of reference ions, injected online during each run, to assess mass accuracy, signal stability and chromatographic consistency across runs. Regular injection of pooled QC samples (run every six samples) was used to evaluate analytical reproducibility and feature-level variability. Repeated injections (replicates) allowed assessment of the injection and measurement variability. Blank controls were used to identify background signal.

### Data processing and analysis

The acquired MS data were processed in Mass Hunter Qualitative Analysis (version B.10.00; Agilent Technologies, Santa Clara, CA, USA). The generated mzDATA files were used for feature detection in XCMS (version 3.7.1, https://xcmsonline.scripps.edu). Feature detection was done with CentWave method and characterized by m/z, retention time, and integrated peak intensity. Signal/noise threshold of 6, prefilter peaks of 3, and prefilter intensity of 500 were applied. Only features present in > 2 replicates of each group were retained. Features missing in > 2 replicates within a group were classified as absent for that group. A standalone software MS-LAMP (https://github.com/Gurpreethgnis/MS-LAMP) with integrated *Mycobacterium tuberculosis* (M. tb) Lipidome database “Mtb LipidDB”^5^ and the database of Lipid Metabolites and Pathways Strategy Consortium (LIPID MAPS)^41^ was used for lipids annotation. The m/z values of features detected in XCMS were assigned to negatively charged [M-H]^−^ (glycerophospholipids, saccharolipids, prenol lipids) or singly protonated [M + H]^+^ (fatty acyls, polyketides, glycerolipids) ions with a maximal mass difference of 0.05 m/z. Abundance of each feature (three replicates) was used to calculate mean, median, and maximum intensity values of annotated compounds, peak-to-peak range, and standard deviation in Python (version 3.12) using the pandas and numpy libraries. Author-developed functions were applied for data merging and filtering, ensuring precise selection and integration. Data presented on graphs are mean values. The relative abundance was calculated as the proportion of each individual lipid relative to the total abundance of all lipids within the same subclass, such that the cumulative abundance of the subclass equals 100%.

PCA. Principal component analysis (PCA) was performed on a feature matrix comprising mean signal intensities for 41 lipid features measured across 13 samples. To mitigate the broad dynamic range of intensities, values were log10-transformed and then autoscaled (z-score standardization; mean-centered and divided by the standard deviation of each variable) so that all lipids contributed comparably to the multivariate model.

Statistics. Group-wise differences in lipid class abundance were assessed using the Kruskal–Wallis test on log10-transformed values, followed by Benjamini–Hochberg false discovery rate (FDR) correction to account for multiple testing. Non-parametric statistical tests were selected due to non-normal data distributions and the limited number of clinical isolates within individual resistance groups.

All analyses were performed in Python 3.13 using standard scientific computing libraries (NumPy, pandas, SciPy, scikit-learn, statsmodels) and Matplotlib for visualization.

## Supplementary Information

Below is the link to the electronic supplementary material.

Supplementary Material 1

## Data Availability

The data that support the findings of this study are available from Zenodo, DOI: 10.5281/zenodo.17214535.

## References

[1] Batt, S. M., Minnikin, D. E. & Besra, G. S. The thick waxy coat of mycobacteria, a protective layer against antibiotics and the host’s immune system. *Biochem. J.* **477** (10), 1983–2006. 10.1042/BCJ20200194 (2020). (From NLM Medline). 32470138 10.1042/BCJ20200194 PMC7261415

[2] Layre, E., Al-Mubarak, R. & Belisle, J. T. Branch Moody, D. Mycobacterial Lipidomics. *Microbiol. Spectr.* **2** (3). 10.1128/microbiolspec (2014). (MGM2-0033-2013 From NLM Medline). 10.1128/microbiolspec.MGM2-0033-2013 26103971

[3] Bansal-Mutalik, R. & Nikaido, H. Mycobacterial outer membrane is a lipid bilayer and the inner membrane is unusually rich in diacylphosphatidylinositol dimannosides. *PNAS* **111**, 4958–4963 (2014). 24639491 10.1073/pnas.1403078111 PMC3977252

[4] Jackson, M. The mycobacterial cell envelope-lipids. *Cold Spring Harb Perspect. Med.* **4** (10). 10.1101/cshperspect.a021105 (2014). From NLM Medline. 10.1101/cshperspect.a021105 PMC4200213 25104772

[5] Sartain, M. J., Dick, D. L., Rithner, C. D., Crick, D. C. & Belisle, J. T. Lipidomic analyses of *Mycobacterium tuberculosis* based on accurate mass measurements and the novel Mtb LipidDB. *J. Lipid Res.* **5**, 861–672. 10.1194/jlr.M010363 (2011). 10.1194/jlr.M010363 PMC3073466 21285232

[6] Nguyen, P. P., Kado, T., Prithviraj, M., Siegrist, M. S. & Morita, Y. S. Inositol acylation of phosphatidylinositol mannosides: a rapid mass response to membrane fluidization in mycobacteria. *J. Lipid Res.* **63** (9), 100262. 10.1016/j.jlr.2022.100262 (2022). 35952902 10.1016/j.jlr.2022.100262 PMC9490103

[7] Angala, S. K., Belardinelli, J. M., Huc-Claustre, E., Wheat, W. H. & Jackson, M. The cell envelope glycoconjugates of Mycobacterium tuberculosis. *Crit. Rev. Biochem. Mol. Biol.* **49** (5), 361–399. 10.3109/10409238.2014.925420 (2014). 24915502 10.3109/10409238.2014.925420 PMC4436706

[8] Queiroz, A. & Riley, L. W. Bacterial immunostat: Mycobacterium tuberculosis lipids and their role in the host immune response. *Rev. Soc. Bras. Med. Trop.* **50** (1), 9–18. 10.1590/0037-8682-0230-2016 (2017). 28327797 10.1590/0037-8682-0230-2016

[9] Ghazaei, C. Mycobacterium tuberculosis and lipids: Insights into molecular mechanisms from persistence to virulence. *J. Res. Med. Sci.* **23** (63). 10.4103/jrms.JRMS_904_17 (2018). From NLM PubMed-not-MEDLINE. 10.4103/jrms.JRMS_904_17 PMC6091133 30181745

[10] Ishikawa, E. et al. Direct recognition of the mycobacterial glycolipid, trehalose dimycolate, by C-type lectin Mincle. *J. Exp. Med.* **206** (13), 2879–2888. 10.1084/jem.20091750 (2009). 20008526 10.1084/jem.20091750 PMC2806462

[11] Augenstreich, J. & Briken, V. Host cell targets of released lipid and secreted protein effectors of *Mycobacterium tuberculosis*. *Front. Cell. Infect. Microbiol.* **10**, 595029. 10.3389/fcimb.2020.595029 (2020). 33194845 10.3389/fcimb.2020.595029 PMC7644814

[12] Takimoto, H. et al. Interferon-gamma independent formation of pulmonary granuloma in mice by injections with trehalose dimycolate (cord factor), lipoarabinomannan and phosphatidylinositol mannosides isolated from *Mycobacterium tuberculosis*. *Clin. Exp. Immunol.* **144** (1), 134–141. 10.1111/j.1365-2249.2006.03043.x (2006). 16542375 10.1111/j.1365-2249.2006.03043.x PMC1809632

[13] Reed, M. B. et al. A glycolipid of hypervirulent tuberculosis strains that inhibits the innate immune response. *Nature* **431** (7004), 84–87. 10.1038/nature02837 (2004). 15343336 10.1038/nature02837

[14] Brodin, P. et al. High content phenotypic cell-based visual screen identifies *Mycobacterium tuberculosis* acyltrehalose-containing glycolipids involved in phagosome remodeling. *PLoS Pathog*. **6** (9), e1001100. 10.1371/journal.ppat.1001100 (2010). 20844580 10.1371/journal.ppat.1001100 PMC2936551

[15] Lee, K. S. et al. Diacyltrehalose of *Mycobacterium tuberculosis* inhibits lipopolysaccharide- and mycobacteria-induced proinflammatory cytokine production in human monocytic cells. *FEMS Microbiol. Lett.* **267** (1), 121–128. 10.1111/j.1574-6968.2006.00553.x (2007). 17156119 10.1111/j.1574-6968.2006.00553.x

[16] Saavedra, R., Segura, E., Leyva, R., Esparza, L. A. & Lopez-Marin, L. M. Mycobacterial di-O-acyl-trehalose inhibits mitogen- and antigen-induced proliferation of murine T cells in vitro. *Clin. Diagn. Lab. Immunol.* **8** (6), 1081–1088. 10.1128/CDLI.8.6.1-91-1088.2001 (2001). 11687444 10.1128/CDLI.8.6.1081-1088.2001 PMC96230

[17] Osman, M. M., Pagan, A. J., Shanahan, J. K. & Ramakrishnan, L. Mycobacterium marinum phthiocerol dimycocerosates enhance macrophage phagosomal permeabilization and membrane damage. *PLoS One*. **15** (7), e0233252. 10.1371/journal.pone.0233252 (2020). 32701962 10.1371/journal.pone.0233252 PMC7377490

[18] Rens, C., Chao, J. D., Sexton, D. L., Tocheva, E. I. & Av-Gay, Y. Roles for phthiocerol dimycocerosate lipids in *Mycobacterium tuberculosis* pathogenesis. *Microbiol. (Reading)*. **167** (3). 10.1099/mic.0.001042 (2021). 10.1099/mic.0.001042 33629944

[19] Wang, H., Liu, D. & Zhou, X. Effect of mycolic acids on host immunity and lipid metabolism. *Int. J. Mol. Sci.* **25** (1). 10.3390/ijms25010396 (2023). 10.3390/ijms25010396 PMC10778799 38203570

[20] Larrouy-Maumus, G. et al. Cell-envelope remodeling as a determinant of phenotypic antibacterial tolerance in *Mycobacterium tuberculosis*. *ACS Infect. Dis.* **2** (5), 352–360. 10.1021/acsinfecdis.5b00148 (2016). 27231718 10.1021/acsinfecdis.5b00148 PMC4877114

[21] Menon, A. P., Lee, T. H., Aguilar, M. I. & Kapoor, S. Decoding the role of mycobacterial lipid remodelling and membrane dynamics in antibiotic tolerance. *Chem. Sci.* **15** (45), 19084–19093. 10.1039/d4sc06618a (2024). From NLM Publisher. 39483253 10.1039/d4sc06618a PMC11520350

[22] Block, A. M. et al. *Mycobacterium tuberculosis* requires the outer membrane lipid phthiocerol dimycocerosate for starvation-induced antibiotic tolerance. *mSystems*. **8**(1), e0069922. 10.1128/msystems.00699-22 (2023). 10.1128/msystems.00699-22 PMC9948706 36598240

[23] Charalambous, K., Miller, D., Curnow, P. & Booth, P. J. Lipid bilayer composition influences small multidrug transporters. *BMC Biochem.* **9**, 31. 10.1186/1471-2091-9-31 (2008). 19032749 10.1186/1471-2091-9-31 PMC2605743

[24] Liu, J. et al. Mutations in Efflux Pump Rv1258c (Tap) Cause Resistance to Pyrazinamide, Isoniazid, and Streptomycin in *M. tuberculosis*. *Front. Microbiol.* **10**, 216. 10.3389/fmicb.2019.00216 (2019). 30837962 10.3389/fmicb.2019.00216 PMC6389670

[25] Sharma, S. et al. Piperine as an inhibitor of Rv1258c, a putative multidrug efflux pump of *Mycobacterium tuberculosis*. *J. Antimicrob. Chemother.* **65** (8), 1694–1701. 10.1093/jac/dkq186 (2010). 20525733 10.1093/jac/dkq186

[26] Brammachary, U. et al. Mechanisms and action of drug resistance on *Mycobacterium tuberculosis*. In *New Insights in Antibiotic Resistance and Pneumococcal Vaccines* (eds Mustafa, G. & Saxena, S. K.) (IntechOpen, 2022).

[27] Carel, C. et al. Mycobacterium tuberculosis proteins involved in mycolic acid synthesis and transport localize dynamically to the old growing pole and septum. *PLoS One*. **9** (5), e97148. 10.1371/journal.pone.0097148 (2014). From NLM Medline. 24817274 10.1371/journal.pone.0097148 PMC4016276

[28] Seidel, M., Alderwick, L. J., Sahm, H., Besra, G. S. & Eggeling, L. Topology and mutational analysis of the single Emb arabinofuranosyltransferase of Corynebacterium glutamicum as a model of Emb proteins of Mycobacterium tuberculosis. *Glycobiology* **17** (2), 210–219. 10.1093/glycob/cwl066 (2007). From NLM Medline. 17088267 10.1093/glycob/cwl066

[29] Murase, L. S. et al. Possible binding of piperine in *Mycobacterium tuberculosis* RNA polymerase and rifampin synergism. *Antimicrob. Agents Chemother.* **63** (11). 10.1128/AAC.02520-18 (2019). 10.1128/AAC.02520-18 PMC6811433 31481438

[30] Campbell, E. A. et al. Structural mechanism for rifampicin inhibition of bacterial rna polymerase. *Cell* **104** (6), 901–912. 10.1016/s0092-8674(01)00286-0 (2001). 11290327 10.1016/s0092-8674(01)00286-0

[31] Jankowski, G. et al. Molecular insight into thymoquinone mechanism of action against *Mycobacterium tuberculosis*. *Front. Microbiol.* **15**, 1353875. 10.3389/fmicb.2024.1353875 (2024). 38414774 10.3389/fmicb.2024.1353875 PMC10896893

[32] Yelamanchi, S. D. et al. Rifampicin-Mediated Metabolic Changes in Mycobacterium tuberculosis. *Metabolites*. **12** (6). 10.3390/metabo12060493 (2022). 10.3390/metabo12060493 PMC9228056 35736426

[33] Jackson, M., Crick, D. C. & Brennan, P. J. Phosphatidylinositol is an essential phospholipid of mycobacteria. *J. Biol. Chem.* **275**, 30092–30099 (2000). 10889206 10.1074/jbc.M004658200

[34] Frallicciardi, J., Melcr, J., Siginou, P., Marrink, S. J. & Poolman, B. Membrane thickness, lipid phase and sterol type are determining factors in the permeability of membranes to small solutes. *Nat. Commun.* **13** (1), 1605. 10.1038/s41467-022-29272-x (2022). From NLM Medline. 35338137 10.1038/s41467-022-29272-x PMC8956743

[35] Slayden, R. A. & Barry, C. E. 3 The role of KasA and KasB in the biosynthesis of meromycolic acids and isoniazid resistance in Mycobacterium tuberculosis. *Tuberculosis (Edinb)*. **82** (4–5), 149–160. 10.1054/tube.2002.0333 (2002). From NLM Medline. 12464486 10.1054/tube.2002.0333

[36] Augenstreich, J. et al. Phthiocerol dimycocerosates from mycobacterium tuberculosis increase the membrane activity of bacterial effectors and host receptors. *Front. Cell. Infect. Microbiol.* **10**, 420. 10.3389/fcimb.2020.00420 (2020). From NLM Medline. 32923411 10.3389/fcimb.2020.00420 PMC7456886

[37] Camacho, L. R. et al. Analysis of the phthiocerol dimycocerosate locus of *Mycobacterium tuberculosis*. Evidence that this lipid is involved in the cell wall permeability barrier. *J. Biol. Chem.* **276** (23), 19845–19854. 10.1074/jbc.M100662200 (2001). 11279114 10.1074/jbc.M100662200

[38] Kirksey, M. A. et al. Spontaneous phthiocerol dimycocerosate-deficient variants of *Mycobacterium tuberculosis* are susceptible to gamma interferon-mediated immunity. *Infect. Immun.* **79** (7), 2829–2838. 10.1128/IAI.00097-11 (2011). From NLM Medline. 21576344 10.1128/IAI.00097-11 PMC3191967

[39] Mulholland, C. V. et al. M. The PDIM paradox of Mycobacterium tuberculosis: new solutions to a persistent problem. *bioRxiv* (2023). 10.1101/2023.10.16.562559.

[40] SambandanD et al. Keto-mycolic acid-dependent pellicle formation confers tolerance to drug-sensitive *Mycobacterium tuberculosis*. *mBio*. **4**10.1128/mBio.00222-13 (2013). 10.1128/mBio.00222-13 PMC3663190 23653446

[41] Sabareesh, V. & Singh, G. Mass spectrometry based lipid(ome) analyzer and molecular platform: a new software to interpret and analyze electrospray and/or matrix-assisted laser desorption/ionization mass spectrometric data of lipids: a case study from *Mycobacterium tuberculosis*. *J. Mass. Spectrom.* **48** (4), 465–477. 10.1002/jms.3163 (2013). From NLM Medline. 23584940 10.1002/jms.3163

