# Supplementary material for: Linking lipid profile alterations to antibiotic tolerance and natural product synergy in drug-resistant Mycobacterium tuberculosis clinical isolates
Source: Sci Rep. 2026 Feb 28;16:11459. doi: 10.1038/s41598-026-41967-5 (PMC13057168; doi:10.1038/s41598-026-41967-5)
Supplement: Supplementary file 1 — Supplementary Material 1 [file 41598_2026_41967_MOESM1_ESM.docx]

**Supplementary file**

Linking Lipid Profile Alterations to Antibiotic Tolerance and Natural Product Synergy in Drug-Resistant *Mycobacterium tuberculosis* Clinical Isolates

Anna Zabost^1^, Rafał Sawicki^2^, Grzegorz Jankowski^2^, Marcin Ziomek^3^, Wiesław Truszkiewicz^2^, Arkadiusz Syta^4^, Benita Hryć^3^, Ewa Augustynowicz-Kopeć^1^, Piotr Podlasz^5^, Małgorzata Chmielewska^5^, Elwira Sieniawska^3*^

^1^ Department of Microbiology, National Tuberculosis and Lung Diseases Research Institute, Warsaw, Poland

^2^ Chair and Department of Biochemistry and Biotechnology, Medical University of Lublin, Lublin, Poland

^3^ Department of Natural Products Chemistry, Medical University of Lublin, Lublin, Poland

^4^ Department of Technical Computer Science, Lublin University of Technology, Lublin, Poland

^5^ Department of Pathophysiology, Forensic Veterinary Medicine and Administration, Faculty of Veterinary Medicine, University of Warmia and Mazury in Olsztyn, Poland

^*^Address correspondence to Elwira Sieniawska, Department of Natural Products Chemistry, Medical University of Lublin, Chodźki 1, 20-093 Lublin, Poland; esieniawska@pharmacognosy.org


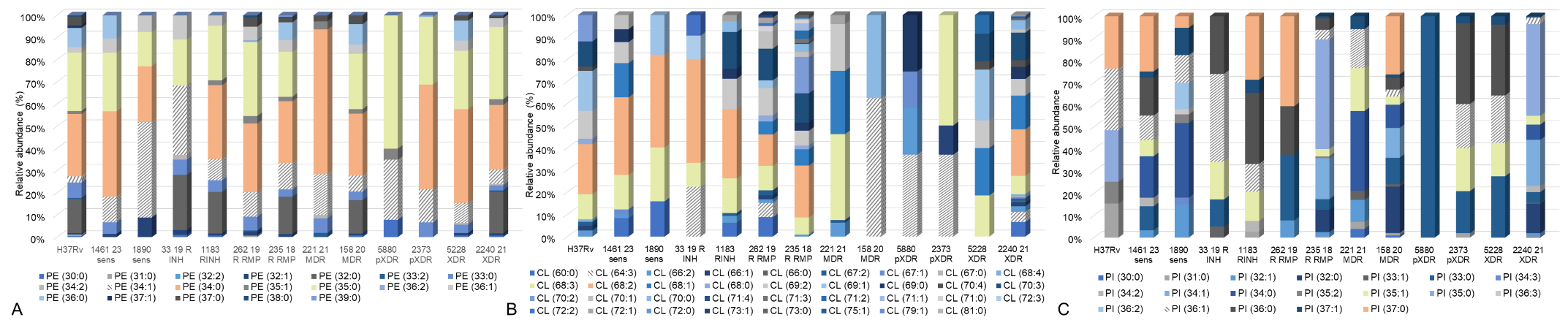


Figure S1. Glycerophospholipids contributing to *Mycobacterium tuberculosis* inner membrane composition. (A) Phosphatidylethanolamine (PE) composition across the analyzed strains. (B) Cardiolipin (CL) composition across the analyzed strains. (C) Phosphatidylinositol (PI) composition across the analyzed strains. Relative abundance is expressed as the proportion of each individual lipid species relative to the total abundance of all lipids within the same subclass, such that the cumulative abundance of each subclass equals 100%.


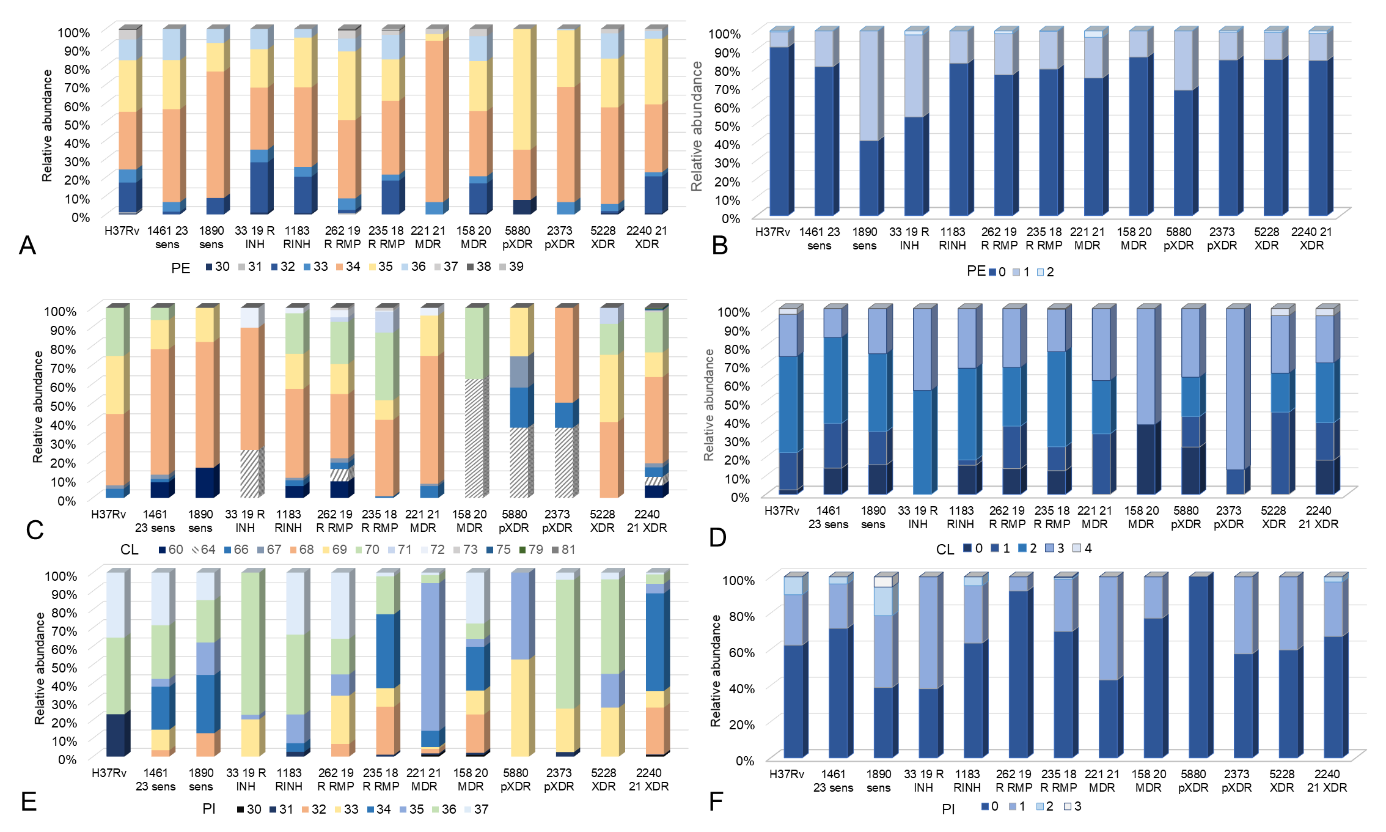


Figure S2. Glycerophospholipid composition across the analyzed Mycobacterium tuberculosis strains. (A) Diacylglycerophosphoethanolamines (PE) grouped by total number of carbon atoms. (B) PE grouped by number of double bonds in the acyl chains. (C) Cardiolipins (CL) grouped by total number of carbon atoms. (D) CL grouped by number of double bonds in the acyl chains. (E) Diacylglycerophosphoinositols (PI) grouped by total number of carbon atoms. (F) PI grouped by number of double bonds in the acyl chains. Relative abundance is expressed as the proportion of each individual lipid species relative to the total abundance of all lipids within the same subclass, such that the cumulative abundance of each subclass equals 100%.


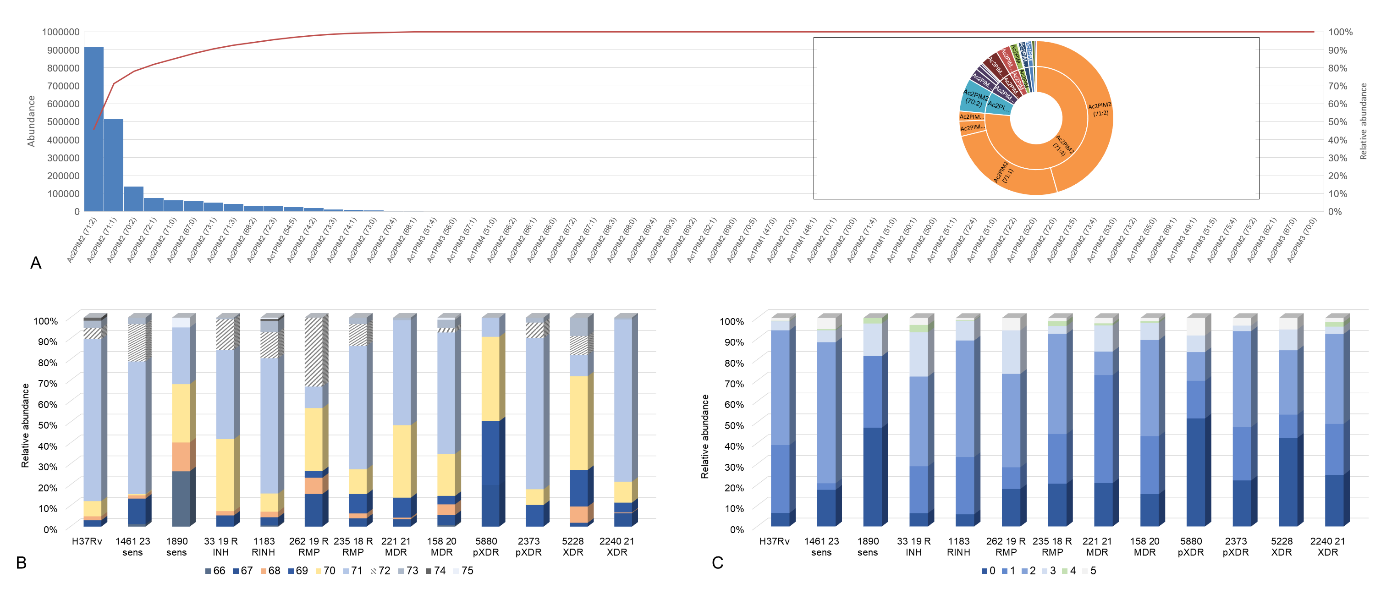


Figure S3. Acylated diacylglycerophosphoinositol mannoside (AcPIM) composition across the analyzed *Mycobacterium tuberculosis* strains. (A) AcPIM species ranked according to their contribution to the total AcPIM subclass. (B) AcPIMs grouped by total number of carbon atoms in the acyl chains. (C) AcPIMs grouped by number of double bonds in the acyl chains. Relative abundance is expressed as the proportion of each individual lipid species relative to the total abundance of all AcPIMs, such that the cumulative abundance of the subclass equals 100%.


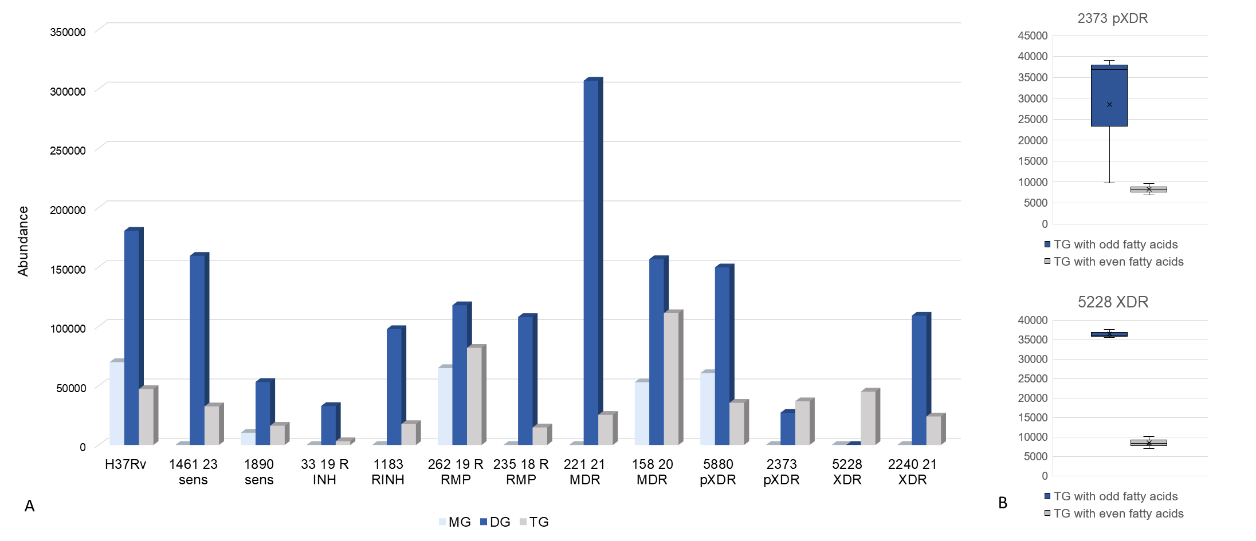


Figure S4. Glycerolipids in the panel strains. A – the abundance and the ratio between glycerolipids in subclasses; B – the abundance of TG with odd and even fatty acids in 2373 pXDR and 5228 XDR isolates. MG – monoacylglicerols; DG – diacylglicerols; TG – triacylglicerols.


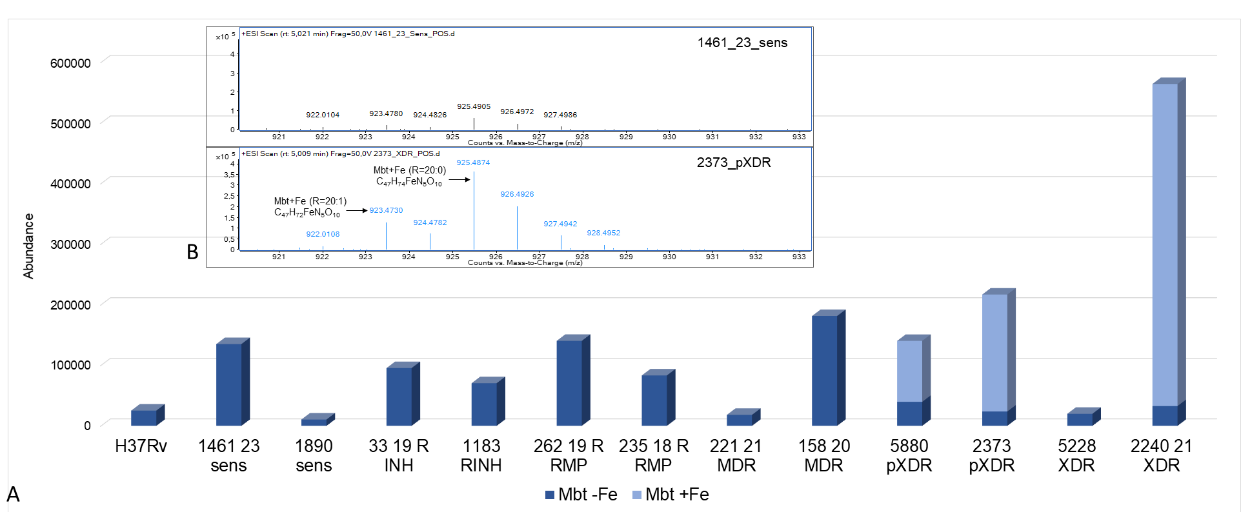


Figure S5. Abundance of mycobactins in the panel strains. Mtb-Fe – mycobactins without iron; Mtb+Fe – mycobactins with trapped iron; B – mass spectra of Mtb+Fe in 1461 23 sensitive and 2373 pXDR strains.

**
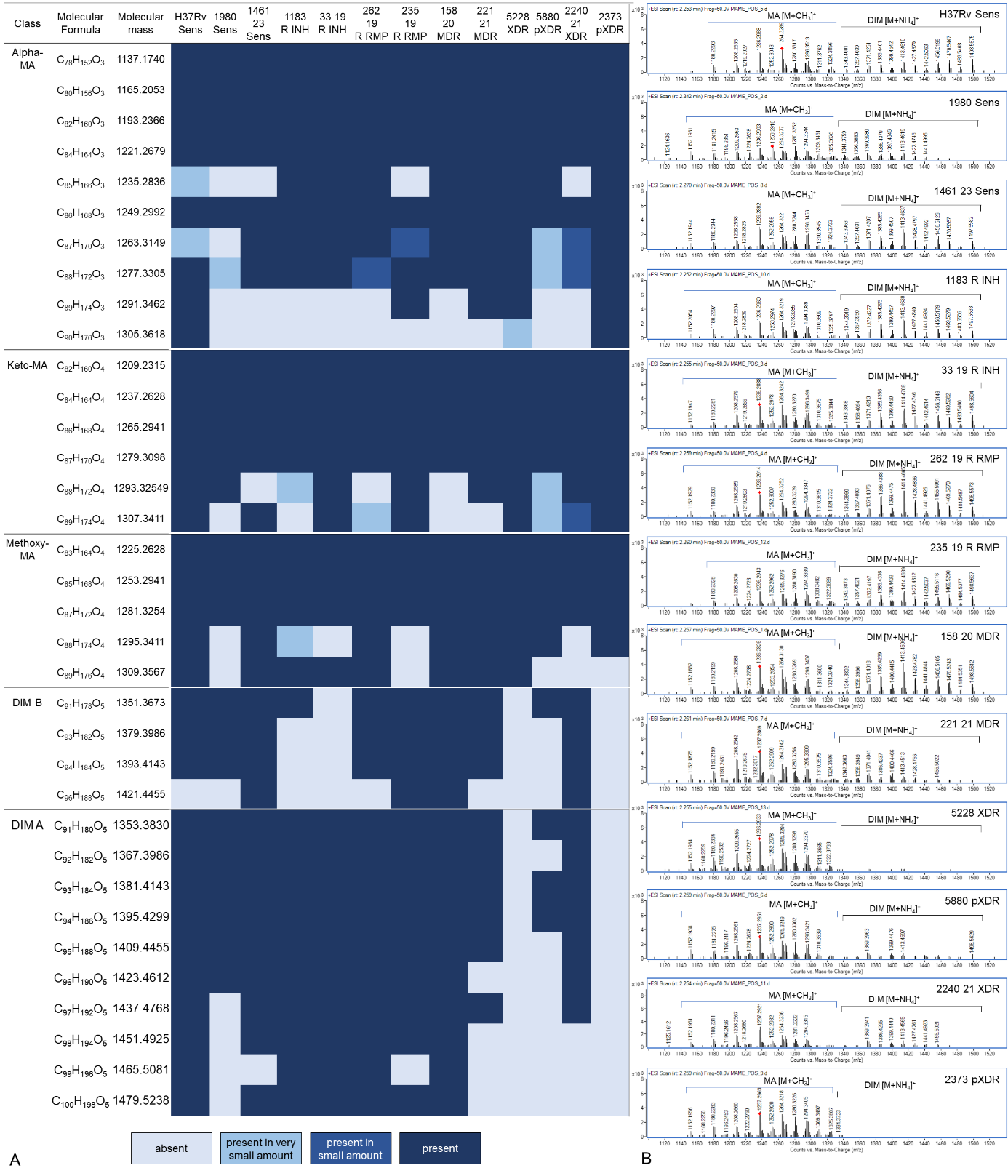
**

Figure S6. Mycolic acids (MA), phthiocerol dimycocerosates (DIMA) and phthiodiolone dimycocerosates (DIMB) in the panel strains. A – presence of alpha-MA, keto-MA, methoxy-MA and DIMA/B; B – mass spectra of MA and DIM observed in panel strains as methylated molecules or as ammonium adducts.
